# Involvement of promoter methylation in the regulation of Pregnane X receptor in colon cancer cells

**DOI:** 10.1186/1471-2407-11-81

**Published:** 2011-02-22

**Authors:** Wataru Habano, Toshie Gamo, Jun Terashima, Tamotsu Sugai, Koki Otsuka, Go Wakabayashi, Shogo Ozawa

**Affiliations:** 1 Department of Pharmacodynamics and Molecular Genetics, School of Pharmacy, Iwate Medical University, 2-1-1 Nishitokuta, Yahaba-Cho, Shiwa-Gun 028-3694, Japan; 2 Division of Molecular Diagnostic Pathology, Department of Pathology, School of Medicine, Iwate Medical University, Iwate Medical University, 19-1 Uchimaru, Morioka 020-8505, Japan; 3 Department of Surgery, School of Medicine, Iwate Medical University, Iwate

## Abstract

**Background:**

Pregnane X receptor (PXR) is a key transcription factor that regulates drug metabolizing enzymes such as cytochrome P450 (CYP) 3A4, and plays important roles in intestinal first-pass metabolism. Although there is a large inter-individual heterogeneity with intestinal CYP3A4 expression and activity, the mechanism driving these differences is not sufficiently explained by genetic variability of PXR or CYP3A4. We examined whether epigenetic mechanisms are involved in the regulation of PXR/CYP3A4 pathways in colon cancer cells.

**Methods:**

mRNA levels of PXR, CYP3A4 and vitamin D receptor (VDR) were evaluated by quantitative real-time PCR on 6 colon cancer cell lines (Caco-2, HT29, HCT116, SW48, LS180, and LoVo). DNA methylation status was also examined by bisulfite sequencing of the 6 cell lines and 18 colorectal cancer tissue samples. DNA methylation was reversed by the treatment of these cell lines with 5-aza-2'-deoxycytidine (5-aza-dC).

**Results:**

The 6 colon cancer cell lines were classified into two groups (high or low expression cells) based on the basal level of PXR/CYP3A4 mRNA. DNA methylation of the CpG-rich sequence of the *PXR *promoter was more densely detected in the low expression cells (Caco-2, HT29, HCT116, and SW48) than in the high expression cells (LS180 and LoVo). This methylation was reversed by treatment with 5-aza-dC, in association with re-expression of PXR and CYP3A4 mRNA, but not VDR mRNA. Therefore, PXR transcription was silenced by promoter methylation in the low expression cells, which most likely led to downregulation of CYP3A4 transactivation. Moreover, a lower level of *PXR *promoter methylation was observed in colorectal cancer tissues compared with adjacent normal mucosa, suggesting upregulation of the PXR/CYP3A4 mRNAs during carcinogenesis.

**Conclusions:**

*PXR *promoter methylation is involved in the regulation of intestinal PXR and CYP3A4 mRNA expression and might be associated with the inter-individual variability of the drug responses of colon cancer cells.

## Background

Nuclear receptor families play a pivotal role in regulating genes involved in drug metabolism and disposition. Pregnane X receptor (PXR, also termed SXR, PAR, and *NR1I2 *as its gene name) is a crucial regulator of various phase I and phase II drug metabolizing enzymes and drug transporters. PXR is expressed in liver, small intestine and other organs. PXR, with a number of therapeutic drugs and other xenobiotics as its ligands, dimerizes with retinoid X receptor α (RXRα). The ligand-PXR-RXRα complex binds to promoter and enhancer elements located upstream of cytochrome P450s (CYPs) 3A and 2C family members, UDP-glucuronosyltransferases (UGTs), sulfotransferases (SULTs), glutathione *S*-transferases (GSTs), and ATP binding cassette (ABC) drug transporters (reviewed in [1-3]).

Wide inter-individual variability has been documented in the expression of hepatic CYP3A4 with respect to basal and PXR-inducible activities. The genetic variability of PXR and CYP3A4 is not sufficiently frequent to explain the apparent inter-individual variability [4]. The inter-individual variability in basal hepatic CYP3A4 expression may include variability in PXR expression, as PXR is activated by endogenous steroid hormones and bile acids. Inter-individual variability of CYP3A4 expression is also observed in human intestine. Interestingly, a report using paired tissue samples of livers and small intestines indicated no observed correlation between the hepatic and small intestine CYP3A4 expression levels [5]. Another research group reported that a majority of CYP3A4 resided in the proximal region of the small intestine, and that the CYP3A4 protein levels decreased dramatically in the distal small intestine [6]. These observations suggest the CYP3A4 is regulated through tissue specific epigenetic regulation in normal tissue. The aim of the present study is to find possible and not yet fully elucidated mechanisms which regulate heterogeneous basal PXR and CYP3A4 expression and activity in cancerous tissues as well. Indeed, several studies previously found a fraction of genes that exhibited inter-individual differences in transcript levels associated with DNA methylation status [7,8].

DNA methylation of the CpG-rich sequence around exon 3 of the *PXR *gene is involved in the epigenetic regulation of PXR in human neuroblastoma [9]. However, epigenetic regulation of PXR and CYP3A4 in human gut is poorly understood. In order to determine whether epigenetic mechanisms function in PXR and CYP3A4 regulation and intestinal metabolism, we examined DNA methylation and mRNA expression of several candidate genes on the PXR/CYP3A4 regulatory pathway in human colon cancer cell lines and tissues.

## Methods

### Cell lines and tissue samples

Human colorectal cancer cell lines LS180, Caco-2, HT29, HCT116, DLD-1, LoVo, SW48 and SW620 were purchased from DS Pharma Biomedical Co., Ltd. (Osaka, Japan). LS180 cells were cultured in E-MEM medium (Invitrogen Corp., Carlsbad, CA) at 37°C under an atmosphere of 5% CO_2_. The other cells were cultured under conditions described elsewhere [10].

Eighteen pairs of cancerous and adjacent normal mucosa were excised from surgical specimens of colorectal cancers. The cancerous and normal epithelia were separated from stroma using crypt isolation [11]. All samples were selected from the same series of cancers as we used in a previous study [10]. All 18 patients with colorectal cancers did not receive chemotherapy before surgical resection. The study protocol was approved by ethics committee of Iwate Medical University (molecular analysis of gastrointestinal tumors and the surrounding mucosa; reference number, H21-140).

### Treatment with 5-aza-2'-deoxycytidine

LS180, LoVo, Caco-2, HCT116, HT29 and SW48 cells were seeded at a concentration of 1 × 10^5 ^cells on a 100 mm dish. The next day, treatment of cells with 0, 0.5 or 5 μM 5-aza-2'-deoxycytidine (5-aza-dC) (Sigma Chemical, St. Louis, MO) was started, and 5-aza-dC was removed by changing the medium 24 h later. The cells were harvested 4 days after removal of 5-aza-dC for DNA and RNA extraction.

### Quantitative real-time PCR analysis of basal CYP3A4, PXR and VDR mRNA levels

Total RNA extraction, cDNA synthesis and real-time PCR were carried out on the cells prepared above, by the same methods as described previously [10]. mRNA levels of the CYP3A4 (exon 3-4, Hs01546612_m1), PXR (exon 5-6, Hs00243666_m1) and vitamin D receptor (VDR) (exon 10-11, Hs01045840_m1) were evaluated by TaqMan Gene Expression Assays (Applied Biosystems, Foster City, CA). In addition, the mRNA level of the PXR splicing variants (exon 1a-2) was also examined by SYBR Green assays using the following primers: 5'-GATTGTTCAAAGTGGACCCC-3'(forward) and 5'-TCCAGGAACAGACTCTGTGT-3'. The mRNA level of the above target genes was normalized to β-actin mRNA [10]. All samples were analyzed in duplicate and average quantities of the gene transcripts were used for calculation. Deviation of the mRNA level of each sample was within 7% of the average.

### DNA methylation analysis of the 6 colon cancer cell lines and 18 colon cancer samples

**Figure 1:**
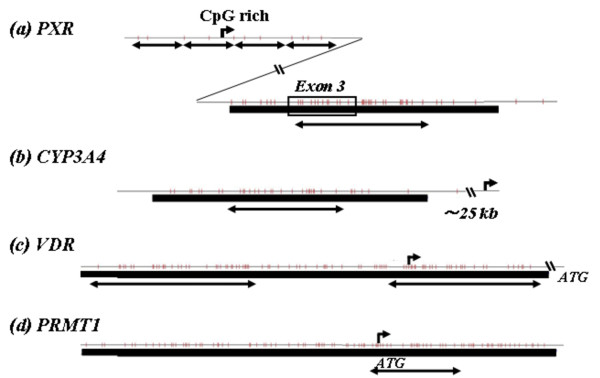
**Location of the CpG sequences examined**. CpG sites (vertical bars), CpG islands identified by CpG island searcher (horizontal thick lines) and transcription start sites within the 5' prime region (curved arrows) are shown in the (a), *PXR*; (b), *CYP3A4*; (c), *VDR*; and (d), *PRMT1 *genes. The segments indicated by double-pointed arrows were examined for DNA methylation by bisulfite direct sequencing.

We found CpG islands within the *PXR *(around exon 3 region), *VDR *(promoter region) and protein arginine methyltrasferase 1(*PRMT1*) (promoter region) genes using the CpG Island Searcher program [12,13]. A CpG island was also detected in the 5' untranslated region (UTR) of the *CYP3A4 *gene (approximately 25 kb distal to the transcription start site). We also found a CpG-rich sequence in the promoter region of the *PXR *gene, although this sequence did not strictly satisfy the criteria for a CpG island [13,14]. The location of all CpG sequences examined in this study are shown in Figure 1. Genomic DNA extracted from the 6 cell lines and the 18 pairs of normal and colon cancer tissue samples was modified by sodium bisulfite, and then each segment including a CpG island or CpG-rich sequence was amplified by PCR and subjected to direct sequencing. The methylation status of the PXR promoter sequence was also estimated by bisulfite sequencing on at least 7 individual DNA strands after subcloning of PCR products into the pCR4-TOPO vector using the TOPO TA Cloning Kit for Sequencing (Invitrogen). A relative methylation level of the PXR promoter was visually determined by the density of each *HpyCH4IV*-digested band using combined bisulfite restriction analysis (COBRA) [15]. The methylation status of the *PXR *exon 3 region was also examined by the COBRA assay using an *HhaI *digestion. All primer sequences are listed in Table 1.

**Table 1:** Primers used for DNA methylation analysis.

Gene	Segment	Primer sequence (5'-3')	Annealing	Product
			temp.(°C)	size (bp)
	1F	GAAGATAATTGTGGTTATTTTTTGGTA	55	574
	1R	CCACCTCCCTAAATAATATTACT		
	
	2F	GTTGTTTTTAGTGGTAAAGGATAGA	55*	602
	2R	CACACACATCTTTTAACTAAAACT		
	
*PXR*	3F	AGGATTTATTATTTTAAGGAGGGGTT	55	230
*promoter*	3R	CCTTAAAACAATACCTCTAACCAT		
	
	4F	GTAAGATTTGGAGATTTTTTATATTTG	55	318
	4R	CTATCCTTCTCTACTAATAAAAATAC		
	
	COBRA	GTAGGGAGAATATAATGAGAATAA	55*	209
		ACTAAAATAAAAACAATACTTCCTCTTCAC		

*PXR*	F	ATTTTTTTATAGGAGGGTTATGAAA	55	292
*exon 3*	R	TACACACRAACACCAACTCACATAT		

*CYP3A4*	F	GAGTTATGGTGGGTTTTATTTAG	55	438
*5'UTR*	R	TCTACATTTCCATCTAAAATACC		

*VDR*	1F	ATAATTTTAGGTTTTAGGAGGTAG	60	400
promoter	1R	CCTAAACTAACCAAACCAAAACTT		
	
	2F	GGGTTGTTTTTGTTTGTTAAAAGG	60	373
	2R	CTTATTACCCAAATACTAAACACT		

*PRMT1*	F	AGGAGAAAGGGGGGGTTTTGGT	55*	273
*promoter*	R	AACCCTTAAAAACTAAAAAACC		

F, forward primer; R, reverse primer

*Dimethyl sulfoxide (5%) was added to the PCR mixture.

## Results

### Basal mRNA levels of the CYP3A4, PXR and VDR genes in the 6 colon cancer cell lines

**Figure 2:**
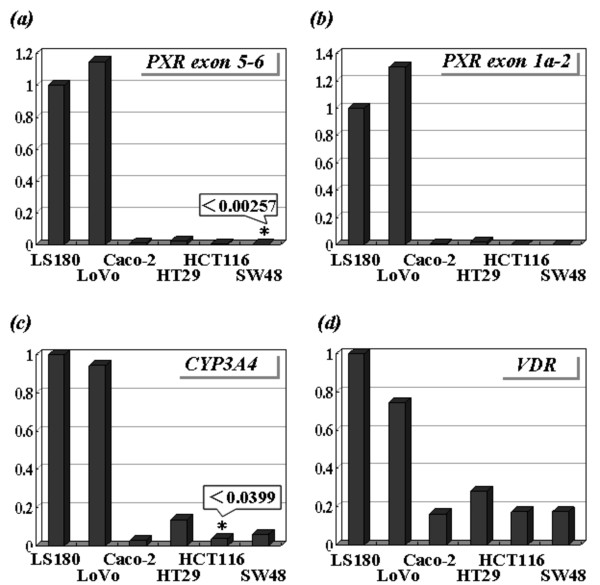
**Basal expression profile of colon cancer cell lines**. Basal levels of (a), PXR exon 5-6; (b), PXR exon 1a-2; (c), CYP3A4; and (d), VDR transcripts in LS180, LoVo, Caco-2, HT29, HCT116 and SW48 cells. The vertical axis indicates a relative transcript level (ratio to LS180 cells). The transcripts indicated by * (PXR exon 5-6 transcript in SW48 cells and CYP3A4 transcript in HCT116 cells) were not detected and we estimated the minimum detectable levels among gradually diluted calibration samples (0.00257 and 0.0399, respectively).

Real-time PCR analyses revealed that the basal levels of CYP3A4, PXR (exon 5-6), PXR (exon 1a-2) and VDR mRNA were heterogeneous among the 6 cell lines examined (Figure 2). The 6 cell lines were then classified into two groups (high or low expression cells) based on the basal level of PXR and CYP3A4 mRNA, because the 6 cell lines always showed either high PXR and CYP3A4 expression or low PXR and CYP3A4 expression. The levels of CYP3A4 and PXR transcripts on high expression cells (LS180 and LoVo) were 7- to 35-fold and 40- to 5,000-fold higher than those on lower expression cells (Caco-2, HT29, HCT116 and SW48), respectively. These two groups also exhibited a difference in the basal level of the VDR transcript, although this difference was not marked (3.5- to 6-fold). There was strong correlation of the levels of the transcripts between PXR (exon 5-6) and PXR (exon 1a-2) throughout all analyses.

### Increased mRNA expression by 5-aza-dC treatment

**Figure 3:**
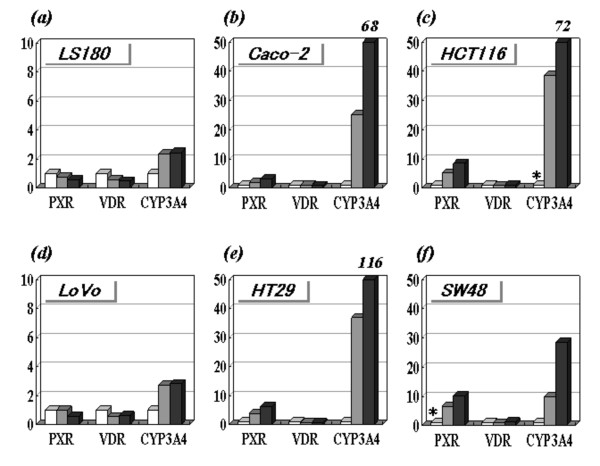
**Expression profile of colon cancer cell lines after 5-aza-dC treatment**. Levels of PXR, VDR and CYP3A4 transcripts in (a), LS180; (b), Caco-2; (c), HCT116; (d), LoVo; (e), HT29; and (f), SW48 cells. Cells were treated with 5-aza-2'-deoxycytidine (0, 0.5 or 5 μM). The vertical axis indicates a relative transcript level (ratio to cells without 5-aza-dC treatment). Numbers *in italics *indicate relative transcript levels that were over the maximum scale on the vertical axis. The transcripts indicated by * (PXR exon 5-6 transcript in SW48 cells and CYP3A4 transcript in HCT116 cells) were not detected and we estimated the minimum detectable levels among gradually diluted calibration samples.

In order to determine whether DNA methylation is involved in the transcriptional regulation of these genes, the 6 cell lines were treated with DNA demethylating agent (5-aza-dC). The treatment with 5-aza-dC induced a clear increase in CYP3A4 (28- to 116-fold) and PXR (3- to 10-fold) transcripts in a dose-dependent manner in the low expression cells, but not in the high expression cells (Figure 3). In particular, the CYP3A4 transcript of the low expression cells eventually reached the levels seen in the high expression cells by 5-aza-dC treatment. In contrast, 5-aza-dC had no marked effect on VDR expression in any of the cell lines (0.5- to 1.2-fold increase). These results suggested that DNA methylation is involved in the regulation of CYP3A4 and PXR, but not VDR, in the low expression cell lines.

### DNA methylation status of the colon cancer cell lines

**Figure 4:**
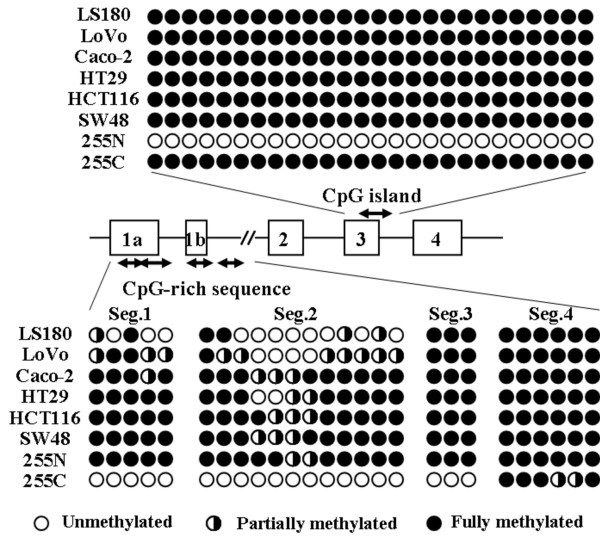
**DNA methylation profile of PXR as detected by direct sequencing**. Methylation status of the *PXR *gene in the 6 colon cancer cell lines and a cancerous tissue sample (255C) and its paired adjacent normal tissue sample (255N) detected by bisulfite direct sequencing. Open and closed circles represent unmethylated and fully methylated CpG sites, respectively. Half-closed circles represent partially methylated CpG sites. The methylation status of the CpG island (around exon 3) and CpG-rich promoter sequence (segments 1, 2, 3 and 4) are shown in the upper and lower panels, respectively.

**Figure 5:**
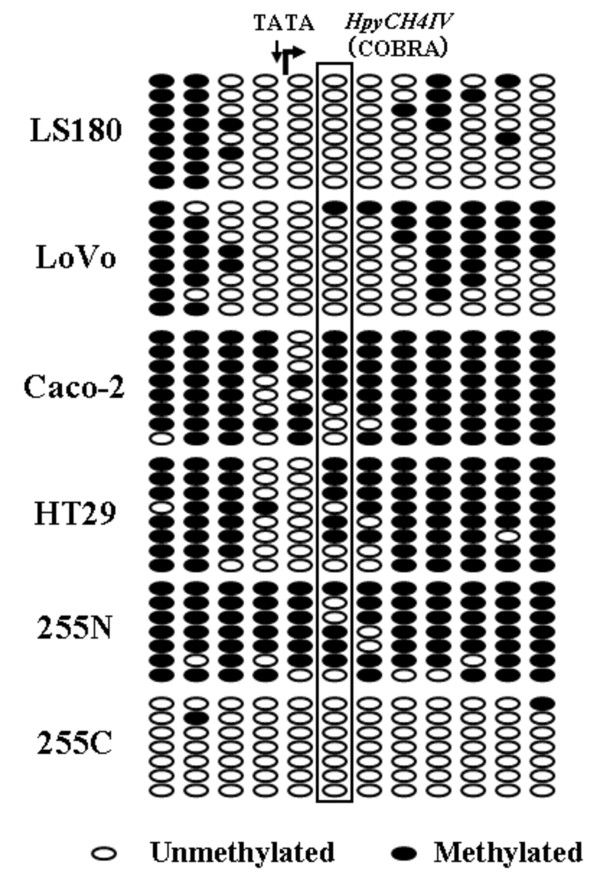
**Detailed DNA methylation profile of PXR segment 2**. Detailed methylation profile of the *PXR *gene in the 6 colon cancer cell lines and a cancerous tissue sample (255C) and its paired adjacent normal tissue sample (255N). Methylation of each CpG site was estimated by bisulfite sequencing on 7 or 8 individual DNA strands after subcloning. Open and closed circles represent unmethylated and methylated CpG sites, respectively. A 'TATA' indicates a putative TATA box. A curved arrow indicates a transcription start site. '*HpyCH4IV*' indicates restriction site using the COBRA assay.

The bisulfite direct sequencing detected no DNA methylation in the *VDR *or *PRMT1 *promoter sequences in the 6 cell lines. Partial methylation of the *CYP3A4 *5'-distal region and full methylation of the *PXR *exon 3 region were equally observed among the 6 cell lines. Therefore, the different expression profiles of the two groups cannot be explained by methylation of these sequences. On the other hand, the CpG-rich sequence of the *PXR *promoter showed a different methylation status among the 6 cell lines. Interestingly, the degree of methylation of the *PXR *promoter (segments 1 and 2) in the high expression cells (LS180 and LoVo) was lower than that in the low expression cells (Caco-2, HT29, HCT116 and SW48) (Figure 4). Therefore, the details of the methylation status of the *PXR *promoter were estimated on individual DNA strands after subcloning (Figure 5). We found that an *HpyCH4IV *site within segment 2 was a suitable marker for the COBRA assay to assess *PXR *promoter methylation, because the degree of methylation of this site showed inverse correlation with the levels of CYP3A4 and PXR expression.

**Figure 6:**
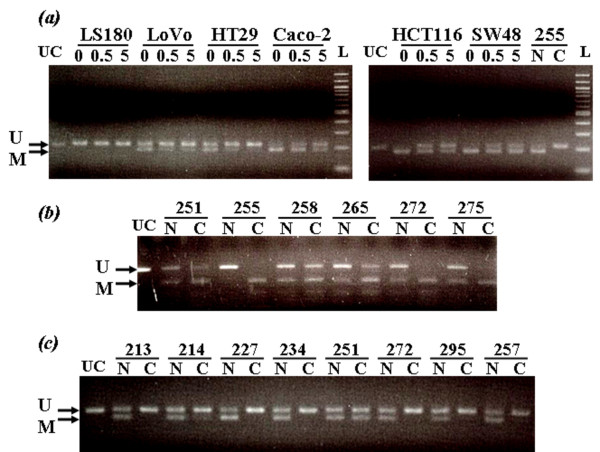
**Methylation status of the *PXR *promoter and exon 3 regions, as detected by the COBRA assay**. Unmethylated and methylated DNAs are shown as U and M, respectively. PCR products that were not cut by the restriction enzymes are shown as UC. (a) *PXR *promoter methylation was examined in the 6 cell lines after treatment with 5-aza-2'-deoxycytidine (0, 0.5 or 5 μM). (b) Methylation status of the CpG island of the *PXR *exon 3 region in 6 of the 18 primary colorectal cancers (numbers are those for particular cases). DNA samples from normal and cancerous tissue are shown as N and C, respectively. Note that a high degree of methylation was detected in cancerous, but not normal, tissue. (c) Methylation status of the CpG-rich sequence of the *PXR *promoter region in 8 primary colorectal cancers. Note that a lower degree of methylation was detected in cancerous tissue compared to normal tissue.

The COBRA assay demonstrated that the treatment with 5-aza-dC resulted in decreased amounts of methylation of the *PXR *promoter in a dose-dependent manner in the low expression cells (Figure 6a). Therefore, the magnitude of methylation was likely to be associated with the decreased levels of *CYP3A4 *and *PXR *gene expression in all 6 cell lines. These results suggest that the *PXR *gene was transcriptionally silenced by methylation of the promoter CpG sites and that the downregulation of the PXR protein resulted in decreased expression of the *CYP3A4 *gene.

The *PXR *promoter methylation was not associated with the profile of microsatellite instability (MSI) or other methylated genes (Table 2). This suggested that altered *PXR *methylation was accumulated during colorectal tumorigenesis, independent of these genetic and epigenetic events.

**Table 2:** Profile of Microsatellite instability (MSI), mismatch repair (MMR) deficiency and promoter methylation in 6 colon cancer cell lines.

		MMR deficiency*		Methylation**		
		
Cell lines	MSI	MLH1	MSH2	*MLH1*	*p16*	*PXR*
LS180	+	+	-	M	?	U
LoVo	+	-	+	U	M	U
Caco-2	-	-	-	?	?	M
HT29	-	-	-	U	M	M
HCT116	+	+	-	U	M	M
SW48	+	+	-	M	M	M

Summary of previous studies [27,28] and the present study (PXR methylation).

*Mismatch repair deficiency was associated with mutations or defects in mRNA transcripts.

**Methylation status was defined as unmethylated (U) or methylated (M).

### DNA methylation status of colon cancer tissue samples

No or slight methylation of the *PXR *exon 3 region was detected in the normal colon tissue samples by direct sequencing and the COBRA assay. The levels of methylation in the cancer tissues were mostly higher than those in the paired adjacent normal tissues (Figure 6b). By contrast, the CpG-rich sequence of the *PXR *promoter was partially methylated in normal tissues, and the degree of methylation was decreased in the paired cancer tissues (Figure 6c). The decreased level of the *PXR *promoter methylation suggested increased expression of the *PXR *gene during colorectal carcinogenesis. There were no differences in the clinicopathological findings between the colorectal cancers with *PXR *methylation and those without methylation.

## Discussion

In the present study, 6 colon cancer cell lines showed heterogeneous mRNA expression profiles and were able to be classified into two groups with respect to their basal levels of the PXR/CYP3A4 transcripts (high expression cells, LS180 and LoVo; low expression cells Caco-2, HT29, HCT116, and SW48). These results are consistent with previous studies, in which LS180 and Caco-2 cells were characterized as PXR-sufficient and PXR-deficient cells, respectively [16,17].

Genetic polymorphisms in the regions that regulate transcription are often a major cause of inter-individual variability in the levels of transcripts. However, such polymorphisms have not been frequently observed in the human *PXR *or *CYP3A4 *genes, implying that certain epigenetic mechanisms are involved in the regulation of PXR and CYP3A4 expression. We found that the CpG-rich sequence within the *PXR *promoter region is methylated to different levels in high and low expression cells. Importantly, the magnitude of this promoter methylation was inversely associated with the levels of PXR and CYP3A4 expression. Furthermore, the levels of the PXR and CYP3A4 transcripts in low expression cells were mostly restored when DNA methylation was reversed by treatment with 5-aza-dC. Although this CpG-rich sequence did not strictly satisfy the criteria for a CpG island, the most affected CpG sites were located in a highly restricted region (segments 1 and 2) and these CpG sites were proximal to several putative transcription factor binding sites (such as Sp1 and hepatocyte nuclear factor 4 alpha) [18-20]. Therefore, *PXR *gene expression is most likely transcriptionally regulated by methylation of these promoter CpG sites.

CYP3A4 is transactivated by functional interplays with VDR-RXRα or PXR-PRMT1 [20-22]. CpG-island methylation of the *VDR *or *PRMT1 *promoter was not detected in these cell lines and the mRNA expression of VDR was not affected by 5-aza-dC treatment. These observations imply that DNA methylation of *PXR*, but not *VDR *or *PRMT1*, resulted in downregulation of the CYP3A4 mRNA in these colon cancer cells.

It is still uncertain whether re-expression of the CYP3A4 by 5-aza-dC treatment was due to the re-expression of some other genes than *PXR*. However, *PXR *must be a candidate for methylation and reduced expression of the PXR by promoter methylation, even if partially, contributes to downregulation of the CYP3A4. Indeed, several studies demonstrated that selective downregulation of the PXR by siRNA reduces the basal level of the CYP3A4 transcripts in a dose-dependent manner [23].

CpG islands in the exon 3 region were fully methylated throughout the cancer cell lines and most cancer tissues. Even after treatment of the cell lines with 5-aza-dC, no increase in the PXR mRNA levels was observed in the high-PXR expressing cell lines, LS180 and LoVo. This strongly suggests that in human colon cancer cells, the methylated CpG islands in the exon 3 play a much less role in the epigenetic regulation of PXR, instead, promoter methylation plays a pivotal role in its regulation. In contrast, Misawa *et al*. previously demonstrated a distinct methylation profile of neuroblastoma cells, in which mRNA expression of the PXR splicing variant (exon 1a-2) was specifically regulated by the methylation of the exon 3 region rather than promoter methylation [9]. We found no marked difference in the levels of the PXR (exon 5-6) and PXR (exon 1a-2) transcripts in the colon cancer cells. Therefore, a tissue-specific DNA methylation profile is most likely involved in the transcriptional regulation of the *PXR *gene.

DNA methylation of the *PXR *promoter was detected in only 1 of the 18 colorectal cancer tissue samples. The results reflect the genuine DNA methylation status, because we examined pure cancerous and normal epithelia using crypt isolation and directly compared the DNA methylation status between paired epithelia. Therefore, a low level of *PXR *promoter methylation, which was observed in the high expression cells, appears to be a common feature of colorectal cancers. We also demonstrated that the level of *PXR *promoter methylation is decreased during carcinogenesis, since paired adjacent normal tissues mostly showed higher levels of *PXR *promoter methylation. We could not directly compare DNA methylation status with the PXR mRNA expression, because crypt isolation provided ethanol-fixed epithelia and it was difficult to obtain fresh mRNA samples. However, most cancer tissues exhibited a pattern of promoter methylation quite similar to that observed in cultured cells with high expression (LS180 and LoVo) (Figures 5 and 6c). Therefore, the association between promoter methylation and transcriptional silencing of the *PXR *gene is most likely applicable to primary colorectal cancers. As observed in the colon cancer cell lines, the decreased level of *PXR *promoter methylation most likely led to increased expression of PXR mRNA in the colorectal cancer tissues. These results are consistent with a recent study that showed strong expression of PXR mRNA in colon cancers, with great variability [24]. In contrast, Ouyang *et al*. found that PXR expression was lost or greatly diminished in many colon cancers using histochemical analysis [25]. Although the role of the altered PXR expression in colorectal carcinogenesis remains to be clarified, Zhou *et al*. demonstrated that PXR plays an antiapoptotic role in colon carcinogenesis by induction of multiple antiapoptotic genes [26].

We cannot rule out the possibility that alterations of the PXR methylation levels play direct roles in tumorigenesis, because certain oncogenes or tumor suppressor genes may be trascriptionally regulated by PXR. Partial methylation of the *PXR *observed in adjacent normal mucosa may be associated with "field defect" for carcinogenesis. However, numerous studies have demonstrated that ligand-binding activation or siRNA-mediated silencing of the PXR can affect the activity of metabolic enzymes including CYP3A4, without changes in the cell proliferation capacity. Therefore, we think that altered level of the *PXR *methylation does not provide a selective growth advantage during colorectal cancer progression.

Interestingly, overexpression of PXR in the colorectal cancer tissue samples was correlated with an increase in UDP glucuronosyl transferases UGT1A1, UGT1A9 and UGT1A10, and led to a marked chemoresistance to the active metabolite of irinotecan (CPT-11) [24]. In addition, CYP3A4 and p-glycoprotein, which are transcriptionally activated by PXR, play important roles in intestinal first-pass metabolism and determine a drug's bioavailability. We hypothesized that PXR may play a key role in the colon cancer cell response to anticancer drugs by modulating expression of drug metabolizing enzymes and transporters including UGT1A, CYP3A4 and p-glycoprotein. Therefore, DNA methylation of the *PXR *promoter might be a good predictor of chemotherapy outcome and toxicity in colorectal cancers.

## Conclusions

*PXR *promoter methylation is involved in the regulation of intestinal PXR and CYP3A4 expression. This methylation might be associated with the inter-individual variability of the drug response of colon cancer cells.

## Abbreviations

PXR: pregnane X receptor; CYPs: cytochrome P450s; VDR: vitamin D receptor; PRMT1: protein arginine methyltrasferase 1; 5-aza-dC: 5-aza-2'-deoxycytidine; COBRA: combined bisulfite restriction analysis

## Competing interests

The authors declare that they have no competing interests.

## Authors' contributions

WH designed this study and carried out the cell culture, molecular genetic studies and drafted the manuscript. GT and JT participated in the data analysis. TS performed crypt isolation and pathological diagnosis. KO and GW performed the surgeries and obtained informed consent from the patients. SO participated in the design of this study and helped to draft the manuscript. All authors read and approved the final manuscript.

## Pre-publication history

The pre-publication history for this paper can be accessed here:

http://www.biomedcentral.com/1471-2407/11/81/prepub
